# Plasma aldosterone is low in patients hospitalized with COVID-19 and not associated with changes in serum potassium levels: *post hoc* observational analyses of clinical trial data

**DOI:** 10.3389/fendo.2025.1706679

**Published:** 2025-12-10

**Authors:** Simon Krenn, Sebastian Hödlmoser, Amelie Kurnikowski, Patrick Jorge, Farsad Eskandary, Georg Heinze, Manfred Hecking, Roman Reindl-Schwaighofer

**Affiliations:** 1 Medical Signal Analysis, Center for Health and Bioresources, AIT Austrian Institute of Technology, Vienna, Austria; 2 Department of Internal Medicine III, Clinical Division of Nephrology and Dialysis, Medical University of Vienna, Vienna, Austria; 3 Department of Epidemiology, Center for Public Health, Medical University of Vienna, Vienna, Austria; 4 Section for Clinical Biometrics, Center for Medical Data Science, Medical University of Vienna, Vienna, Austria

**Keywords:** COVID-19, SARS-CoV-2, aldosterone, renin-angiotensin-aldosterone-system, potassium, hypokalemia

## Abstract

**Background:**

Hypokalemia is common in patients hospitalized with coronavirus disease 2019 (COVID-19) and is associated with mortality, disease progression and severity. Increased aldosterone levels were previously suggested to be the main cause of potassium loss in this population. We aimed to assess the effects of the latest morning plasma aldosterone levels on changes in serum potassium during severe acute respiratory syndrome coronavirus 2 (SARS-CoV-2) infection.

**Methods:**

We used dynamic generalized estimating equations (GEEs) on longitudinal data (3 weeks) from an adequately sized (159 patients) platform trial evaluating therapeutics for COVID-19 during the first wave of infections in Vienna, Austria. We adjusted for important confounding variables (GEE A, 106 patients) and conducted sensitivity analyses by including medications with the potential to confound the analysis (GEE B, 82 patients) and by modeling an exponential decay of effects on potassium over time (GEE C, 82 patients). Furthermore, we explored the relationship descriptively.

**Results:**

The median potassium concentration was 3.8 (quartile 1: 3.5, quartile 3: 4.0) mmol/L, and hypokalemia (<3.5 mmol/L) was present in 15.7% of patients at the first blood draw compared to 21.6% throughout the 3-week observation period. The median aldosterone concentration was 45.0 (20.0, 104.0) pmol/L and was below the lower limit of quantitation (20 pmol/L) in 32.4% of samples. Aldosterone was not associated with changes in potassium neither in GEE A [base-10 logarithm of aldosterone, *β*: −0.008 (95% CI: −0.074, 0.057), *p*-value: 0.805], in GEE B [*β*: 0.013 (−0.064, 0.090), *p*-value: 0.739], nor in GEE C [*β*: 0.001 (−0.078, 0.075), *p*-value: 0.971].

**Conclusion:**

Aldosterone levels were low and were not associated with potassium changes in patients hospitalized with COVID-19 during the first wave of the SARS-CoV-2 pandemic.

## Introduction

Patients with coronavirus disease 2019 (COVID-19) exhibit high rates of hypokalemia (1), which were found to be associated with death or intensive care unit (ICU) admission (2), progression (3, 4) and severity of disease (2, 5–8), prolonged hospital (3, 5) and ICU stay (3), and need for invasive mechanical ventilation (3), as well as mortality risk (9–15). Overall, serum potassium exhibits a J-shaped relationship with risk of death in COVID-19, with severe hyperkalemia being associated with the highest risk (9, 10).

Early in the COVID-19 pandemic, decreased angiotensin-converting enzyme 2 (ACE2) activity after infection, followed by rising aldosterone levels and resulting in urinary potassium loss, was suggested as the primary mechanism for hypokalemia in COVID-19 (7) and has since become a commonly invoked theory when discussing this topic (2, 6, 16–20). Our own research group was among the first to show a positive association of systemic ACE2 with COVID-19 severity, and we also found an overall highly active systemic alternative RAS (21), which ran counter to this originally proposed mechanism. Multiple other studies found that key renin–angiotensin–aldosterone system (RAAS) components remained largely unaffected by the infection (6, 22–24). Histopathological evidence of tubulopathy (25–27) and direct activation of the epithelial Na^+^ channel by the severe acute respiratory syndrome coronavirus 2 (SARS-CoV-2) nucleocapsid (28) further pointed toward different mechanistic explanations. While it was noted early on that “measurement of components of the RAS pathway is necessary to make conclusions about the etiology of hypokalemia in SARS-CoV-2 infection,” (29) no such analysis has thus far been published to our knowledge.

Therefore, we aimed to explore the relationship between aldosterone and potassium changes through 3 weeks of hospital stay in patients with COVID-19.

## Methods

### Study cohort and ethics approval

We retrospectively analyzed data from 159 patients with SARS-CoV-2, hospitalized at a tertiary care site (“Klinik Favoriten” in Vienna, Austria) between 15 March and 31 August 2020. All patients were enrolled in the Austrian Coronavirus Adaptive Clinical Trial (ACOVACT), which was registered with clinicaltrials.gov (ClinicalTrials.gov identification code NCT04351724), and the European Union Drug Regulating Authorities Clinical Trials Database (EudraCT identification code 2020-001302-30). ACOVACT was a multicenter, randomized, active-controlled, open-label platform trial evaluating the efficacy and safety of therapeutics for COVID-19. Patients provided written informed consent to participate, and the study was approved by the Ethics Commission of the Medical University of Vienna (ethics vote identification code 1315/2020). The present study comprises a retrospective, secondary analysis utilizing data from ACOVACT beyond its originally intended use, with permission from the Ethics Commission of the Medical University of Vienna via amendment. All subjects enrolled in ACOVACT were also considered eligible for the present study. The study included consenting, adult patients, hospitalized for COVID-19, requiring oxygen support, with laboratory-confirmed SARS-CoV-2 infection. The exclusion criteria were life expectancy <1 month due to other reasons (e.g., terminal cancer), pregnancy, breastfeeding, unwillingness to receive oral contraceptives (for female patients), severe liver dysfunction (e.g., ALT/AST > 5 times upper limit of normal), stage 4 chronic kidney disease or higher, dialysis, HIV, viral hepatitis, foreseeable hospital discharge within 48 h, allergies, and contraindications to compounds studied in ACOVACT. The detailed inclusion and exclusion criteria of the different subgroups of the trial can be found in the **Supplementary Material**.

### Measurements

Plasma concentrations of aldosterone and angiotensin (Ang) II were determined from EDTA venous blood samples collected up to thrice weekly in the morning in a supine position, centrifuged promptly after collection, and stored on-site at −80°C. They were transferred to a dedicated independent laboratory (Attoquant Diagnostics, Vienna, Austria) and analyzed using liquid chromatography tandem mass spectrometry (LC/MS-MS). Plasma aldosterone concentration was assessed by spiking after 1 h *ex vivo* incubation at 37°C with stable isotope-labeled internal standard for aldosterone and Ang II at concentrations of 1,387.0 pmol/L (500 pg/mL) and 191.4 pmol/L (200 pg/mL), respectively. This procedure was described in more detail in the supplement to a previous publication (30). Serum potassium, sodium, C-reactive protein (CRP), and relevant medication information were retrieved from hospital databases. Hyperkalemia was defined as serum potassium >5 mmol/L, hypokalemia as serum potassium <3.5 mmol/L, hypernatremia as serum sodium >145 mmol/L, and hyponatremia as serum sodium <135 mmol/L. If multiple laboratory values were available for a patient on any given day, we used the first value of that day to best align with our statistical analysis and to reduce the likelihood of acute interventions obscuring the effects of interest. Both arterial and venous measurements were regarded as admissible. The starting point for all analyses was the day of hospitalization or transfer to Klinik Favoriten’s dedicated COVID-19 care facility after testing positive for SARS-CoV-2 at another hospital, and the end point was defined as 21 days past this starting point. Medication information was retrieved *post hoc* from medical records and was defined as having been prescribed an in-class substance on a given day, regardless of dose or route of application. Spot urine sample data for urine potassium-to-creatinine ratio (urine K/Cr ratio) assessment were not originally planned in this study but were ordered per indication at the physician’s discretion and were retrieved *post hoc* from hospital records.

### Statistical analysis

#### Descriptives

Data were presented as median and bounds of the interquartile range (IQR) or number of patients and percentages. For data visualizations, we used bar plots for prevalence rates, scatter plots for bivariate relationships, scatterplot smoothed (LOESS) curves for cohort-level trajectories over time, and line plots for individual trajectories over time. Axes were visually contracted to a logarithmic scale if required for improved visibility and/or interpretability, but the variables and units shown were not transformed.

#### Testing for the effects of aldosterone on potassium levels

Generalized estimating equations (GEEs) were fitted to dynamically model current serum potassium as a dependent variable (DV) on the logarithm of the latest measured plasma aldosterone to the base of 10 as an independent variable (IV). Estimation by GEEs was chosen for simplicity, robustness, interpretability, suitability for longitudinal data with clustering, and efficiency under temporally correlated residuals. We performed complete-case analyses (i.e., at least one datapoint of each variable included in the model, in the proper time configuration as specified below) to avoid imputations based on assumptions that were themselves being tested in this study. For the following analyses, we removed all data of patients who received angiotensin receptor blockers or angiotensin-converting enzyme inhibitors at any time during the hospital stay, as these medications can interfere with the relationship of interest. We performed one main analysis (GEE A) and two sensitivity analyses (GEEs B and C). We adjusted for relevant covariates, described below, in all GEEs (GEEs A, B, and C). In the sensitivity analyses, we additionally included potassium-altering medications as IVs (GEEs B and C) and modeled medication effects to decrease exponentially over time (only GEE C). GEEs were fitted using the R programming language’s geepack library, and the robust sandwich estimator was used to account for clustering on the patient level. Days since hospitalization were used to define a first-order autoregressive intracluster working correlation matrix structure over time. Residuals of all GEEs were assessed for normality. Introducing restricted cubic splines was to be considered per the analysis plan, but the residuals did not indicate the necessity to utilize them.

#### Variables of GEEs A, B, and C

The DV was potassium (continuous in mmol/L) in all three GEEs—A, B, and C. The following IVs and transformations were included in the respective models (IV units are shown in the originally measured units prior to any of the described logarithmic transformations).

IVs of GEE A: age (continuous in decades), sex (binary), currently in ICU (binary), days since hospitalization (continuous in days), base-10 logarithm of the latest (meaning at least 1 day earlier than DV) measured aldosterone level (continuous in pmol/L), interaction of base-10 logarithm of the latest measured aldosterone level with the time interval to DV (continuous in days * pmol/L), latest measured potassium level (continuous in mmol/L), and its interaction with elapsed time to DV (continuous in days * mmol/L). For improved interpretability of the effects, we leveled the time components against the first elapsed day by deducting 1 day from all time intervals.

Additional IVs of GEEs B and C: The same IVs were used as in GEE A, plus potentially potassium-altering medications. These were coded for having received prior to the respective observation of the DV an angiotensin receptor blocker, an angiotensin-converting enzyme inhibitor, a mineralocorticoid receptor antagonist, a loop diuretic, a thiazide-like diuretic, a potassium-flushing drug, potassium supplements, catecholamines during the observation period (each as a separate binary variable), and their respective interactions with the time elapsed between last receiving the medication and the observation of the DV potassium (continuous in days). Only medication classes were encoded, as opposed to single active compounds, and doses could therefore not be considered, because certain compounds were very rare, and we wished to preserve adequate degrees of freedom.

Temporal modification GEE C: To account for a possible exponential decay of the effect of IV aldosterone, the latest potassium level, and medications on the DV potassium levels, we exponentiated the negative days elapsed to the base of 2 to reflect an exponential decrease in these IVs’ effects on the DV in GEE C. We then deducted this time component from 0.5 to again level the comparison against the first elapsed day for improved interpretability of effects (as two to the power of minus one equals 0.5).

#### Assessing the importance of potassium and ICU information when modeling angiotensin II on aldosterone

An originally planned analysis involving linear mixed effects models with the DV aldosterone, comparing Akaike and Bayesian information criteria and using likelihood ratio tests when consecutively adding IVs Ang II, potassium, and the current stay in ICU versus normal ward to a null model, proved unfeasible due to a high rate (32.4%) of aldosterone values below the lower limit of quantitation of 20 pmol/L, as these led to violations of required model assumptions. To replace this analysis, we deviated from the predefined statistical analysis plan and instead fitted GEEs with a sandwich estimator for robustness and compared bias-corrected marginal *R*² as a measure of improvement in captured variance. We used the grouped_bootstraps function from the R programming language’s rsample package to generate 5,000 patient-level bootstrapped datasets, performed marginal *R*² bias correction (by subtracting the difference from the bootstrap mean estimate), and calculated bias-corrected and accelerated (BCa) confidence intervals as implemented in rsample to describe changes in variance capture when adding IVs Ang II, potassium, and ICU information to a null model with the DV aldosterone. Data from patients who received an angiotensin receptor blocker or angiotensin-converting enzyme inhibitor were removed from this analysis.

## Results

### Patient characteristics

The characteristics of all 159 patients, as well as subpopulations, analyzed separately in GEE A and GEEs B and C, are shown in **Table 1**. Patients were predominantly male (66%) and had a median age of 63 years (IQR 50 to 76 years). There was a high prevalence of hypertension (61.6%), diabetes (28.9%), and COPD (10.7%).

**Table 1:** Patient characteristics.

Variable	Overall (*N* = 159)	GEE A (*N* = 106)	GEEs B and C (*N* = 82)
Age (years), median [IQR]	63.0 [50.0, 76.0]	60.5 [47.0, 76.0]	66.0 [55.0, 77.0]
Female, N (%)	54 (34.0%)	33 (31.1%)	24 (29.3%)
BMI (kg/m²), median [IQR]	26.5 [24.5, 30.8]	26.9 [24.8, 31.0]	27.1 [25.4, 30.9]
Missing	81 (50.9%)	36 (34.0%)	31 (37.8%)
Hypertension, *N* (%)	98 (61.6%)	58 (54.7%)	55 (67.1%)
COPD, *N* (%)	17 (10.7%)	11 (10.4%)	10 (12.2%)
Diabetes mellitus, *N* (%)	46 (28.9%)	33 (31.1%)	29 (35.4%)
ICU, *N* (%)	59 (37.1%)	41 (38.7%)	39 (47.6%)
Potassium (mmol/L), median [IQR]	3.8 [3.5, 4.0]	3.8 [3.5, 4.0]	3.8 [3.5, 4.0]
Missing	35 (22.0%)	0 (0%)	0 (0%)
Aldosterone (pmol/L), median [IQR]	45.0 [20.0, 104.0]	39.7 [20.0, 96.1]	41.2 [20.0, 98.3]
Missing	13 (8.2%)	0 (0%)	0 (0%)
Angiotensin II (pmol/L), median [IQR]	73.9 [19.2, 187.0]	65.8 [19.2, 183.0]	56.4 [19.3, 187.0]
Missing	13 (8.2%)	0 (0%)	0 (0%)
CRP (mg/L), median [IQR]	67.2 [33.1, 124.0]	68.9 [33.8, 125.0]	71.3 [40.4, 127.0]
Missing	34 (21.4%)	0 (0%)	0 (0%)

Patient characteristics are presented as median and interquartile range or absolute number and relative percentage of patients. Missingness of data is reported in absolute and relative terms. Presented characteristics include age, female sex, body mass index, diabetes mellitus, hypertension, chronic obstructive pulmonary disease, intensive care unit stay, potassium, aldosterone, angiotensin II, and C-reactive protein. They are reported for all patients and separately for the populations available to the three GEE analyses with the dependent variable potassium performed during the study.

BMI, body mass index; COPD, chronic obstructive pulmonary disease; CRP, C-reactive protein; ICU, intensive care unit; IQR, bounds of the interquartile range.

### Medication

The most prescribed antihypertensives were beta blockers (*N* = 34 patients, 21.4%), followed by angiotensin receptor blockers and calcium channel blockers (both *N* = 24, 15.1%). Loop diuretics were the most common diuretics (*N* = 42, 26.4%), and electrolyte supplementation was especially common for potassium (*N* = 58, 36.5%). The weekly and overall counts of patients receiving medication can be found for each recorded medication class in **Table 2**.

**Table 2:** Medications.

Drug type	Overall *N* (%)	First week *N* (%)	Second week *N* (%)	Third week *N* (%)
Alpha blockers	18 (11.3%)	12 (7.5%)	17 (10.7%)	13 (8.2%)
Alpha-2 adrenoreceptor agonists	6 (3.8%)	4 (2.5%)	1 (0.6%)	2 (1.3%)
Angiotensin-converting enzyme inhibitors	16 (10.1%)	10 (6.3%)	11 (6.9%)	14 (8.8%)
Angiotensin receptor blockers	24 (15.1%)	21 (13.2%)	15 (9.4%)	17 (10.7%)
Beta blockers	34 (21.4%)	28 (17.6%)	25 (15.7%)	25 (15.7%)
Calcium channel blockers	24 (15.1%)	18 (11.3%)	23 (14.5%)	19 (11.9%)
Calcium supplementation	17 (10.7%)	13 (8.2%)	10 (6.3%)	11 (6.9%)
Carbonic anhydrase inhibitors	6 (3.8%)	3 (1.9%)	2 (1.3%)	2 (1.3%)
Catecholamines	31 (19.5%)	30 (18.9%)	24 (15.1%)	16 (10.1%)
Loop diuretics	42 (26.4%)	34 (21.4%)	34 (21.4%)	24 (15.1%)
Magnesium supplementation	40 (25.2%)	31 (19.5%)	21 (13.2%)	15 (9.4%)
Mineralocorticoid receptor antagonists	7 (4.4%)	3 (1.9%)	4 (2.5%)	6 (3.8%)
Phosphate supplementation	34 (21.4%)	24 (15.1%)	22 (13.8%)	13 (8.2%)
Potassium channel openers	0 (0.0%)	0 (0.0%)	0 (0.0%)	0 (0.0%)
Potassium-flushing drugs	9 (5.7%)	6 (3.8%)	2 (1.3%)	1 (0.6%)
Potassium supplementation	58 (36.5%)	47 (29.6%)	34 (21.4%)	25 (15.7%)
Sulfonamide diuretics	6 (3.8%)	3 (1.9%)	5 (3.1%)	2 (1.3%)
Thiazide diuretics	8 (5.0%)	7 (4.4%)	6 (3.8%)	6 (3.8%)

Shown are the numbers of patients receiving an in-class substance during the entire 3-week analysis period and stratified by week. Supplementation includes electrolyte infusions and oral supplementation of both.

### RAAS and potassium dynamics

**Figure 1:**
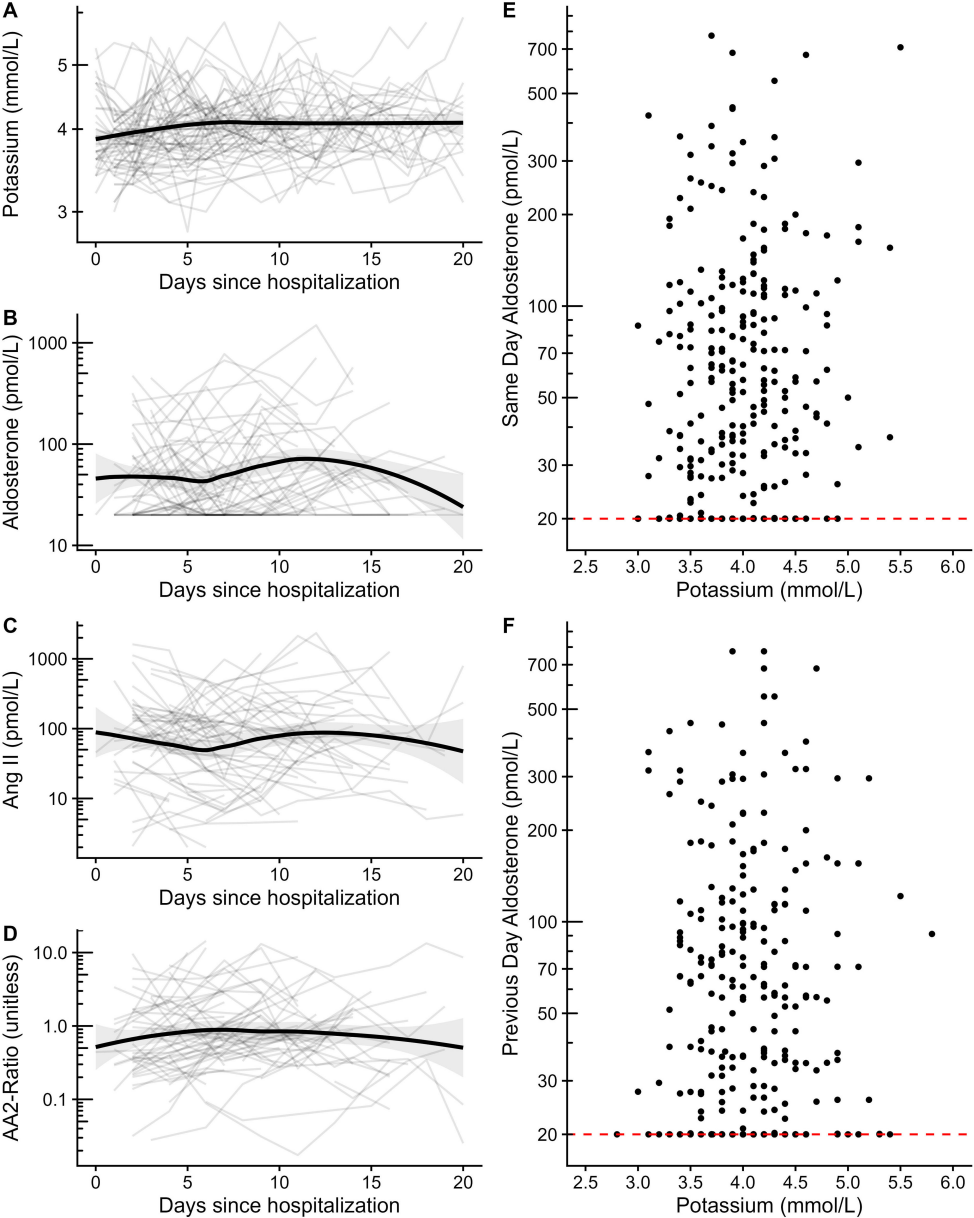
Potassium and aldosterone levels. Trends of mean potassium **(A)**, aldosterone **(B)**, Ang II **(C)**, and aldosterone–angiotensin II ratio **(D)** are depicted through 21 days of hospital care by LOESS smoothed curves with 95% confidence intervals in patients not receiving angiotensin receptor blockers and without ACE inhibitors. Individual patient curves are shown in light gray in the background of these panels. Scatterplots of potassium and aldosterone measured on the same day **(E)** and with aldosterone measured on the previous day **(F)** are shown on the right-hand side. The dashed lines indicate the lower limit of quantitation for aldosterone. RAAS blood samples were drawn in the supine position. All vertical axes were depicted on the logarithmic scale. AA2-Ratio, aldosterone–angiotensin II ratio; Ang, angiotensin.

Aldosterone levels were below the level of quantitation (20 pmol/L) in 32.4% of the samples. The course of potassium, aldosterone, Ang II, and aldosterone–angiotensin-2 ratio (AA2-R) was relatively stable through 21 days of hospital stay on the population level, but more variable in individual patients (**Figures 1A–D**). Hypokalemia, defined as serum potassium below 3.5 mmol/L at any time during the 3-week observation period, occurred in 21.6% of the patients. The population average of potassium remained close to 4 mmol/L during the entire 3-week period. Potassium and aldosterone levels of the current and previous day did not show any clear pattern of association as shown in **Figures 1E, F**. This finding was consistent throughout all 3 weeks of observation, but data were notably sparser during the later weeks of the trial as patients were successively discharged or died (shown on a week-by-week basis in **Supplementary Figure S1** in the **Supplementary Material**).

### Prevalence of potassium and sodium derangements

**Figure 2:**
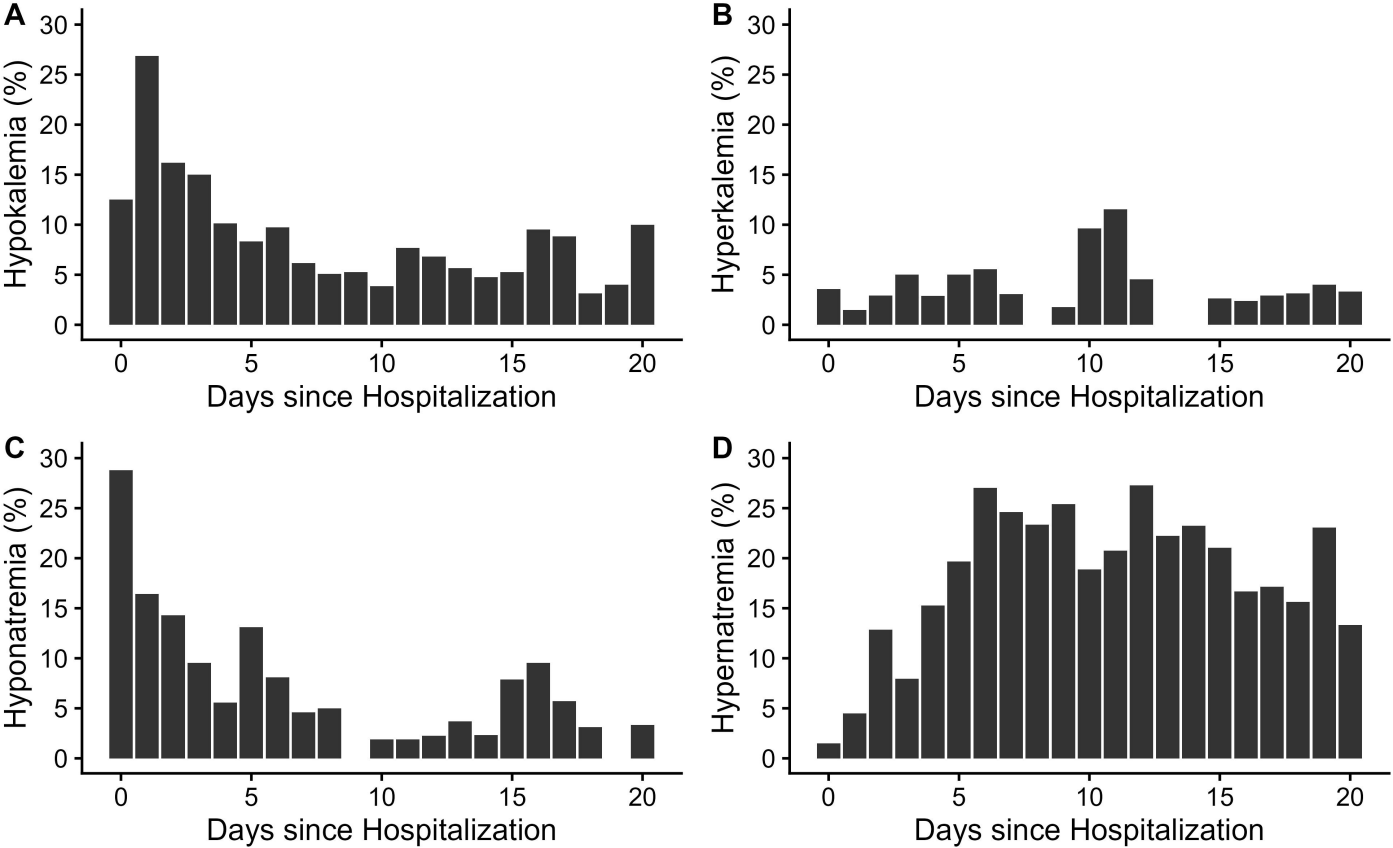
Proportion of blood samples with pathological levels of potassium and sodium per day. Proportions of blood samples with potassium <3.5 mmol/L **(A)**, potassium >5 mmol/L **(B)**, sodium <135 mmol/L **(C)**, and sodium >145 mmol/L **(D)** of total blood samples per day are depicted through 21 days of hospital care.

**Figure 3:**
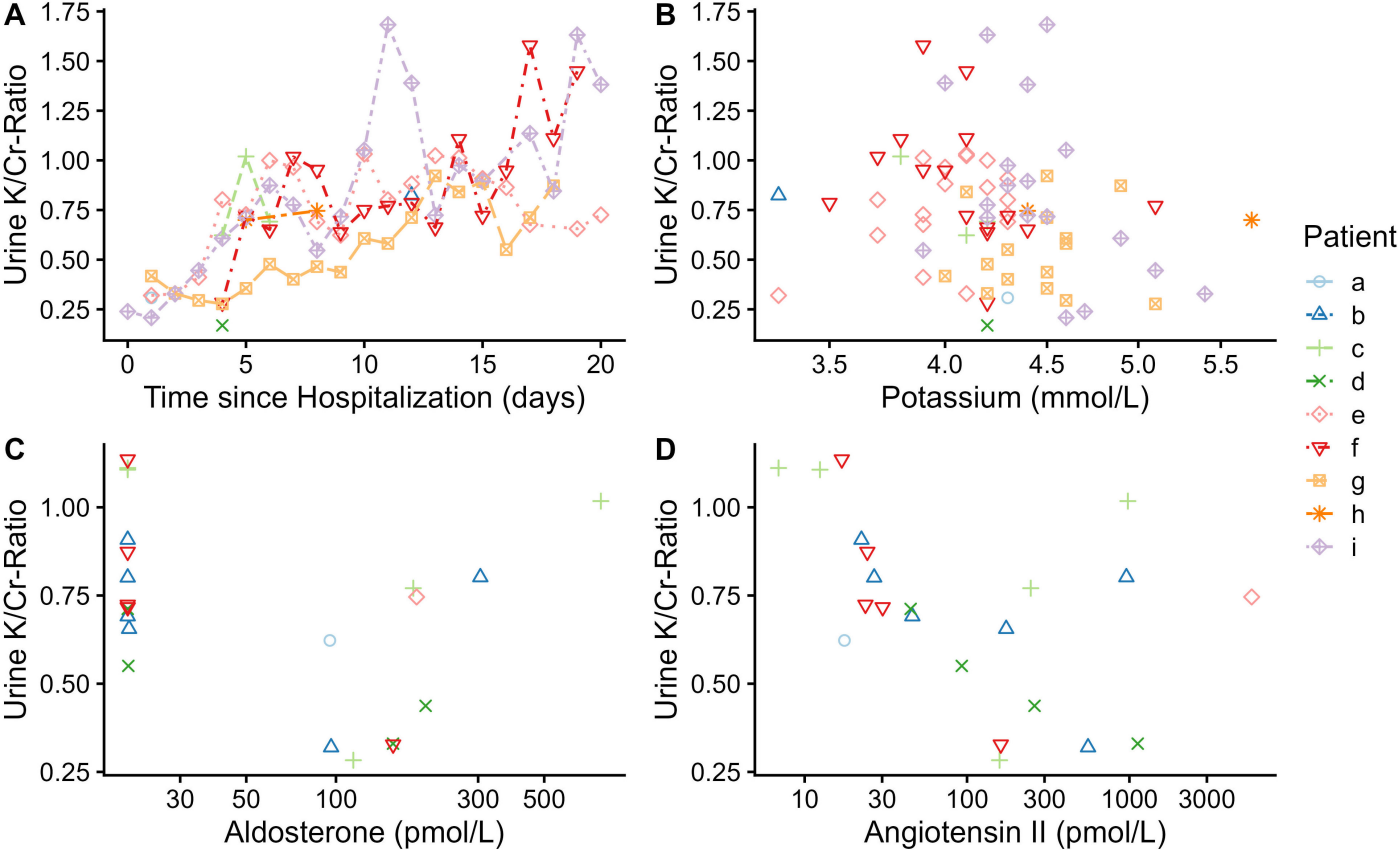
Scatterplots of urine potassium-to-creatinine ratios over time and against potassium, aldosterone, and angiotensin II. Urine potassium–creatinine ratios (which are unitless) in nine patients were line-potted over time **(A)** and scatter-plotted against potassium **(B)**, aldosterone **(C)**, and angiotensin II **(D)** using all data available for the same day. Color, line type, and marker shape indicate each of the nine patients, as seen in the legend on the right side of the plot (letters a–i). Axes for angiotensin and aldosterone were depicted on the logarithmic scale. Cr, creatinine; K, potassium.

In the 3 weeks of hospital stay, relative hypokalemia and hyponatremia prevalence rates were the highest during the first week (the proportion of the first daily blood draws showing hypokalemia or hyponatremia is depicted on a per-day basis in **Figure 2**). At first blood draw of the first week, hypokalemia was found in 17.0% of patients and was almost twice as common in women compared with men (24.1% versus 13.3%, **Table 3**). Conversely, hyponatremia was more common in men compared with women (22.9% versus 9.3%) at this initial blood draw and showed a similar overall prevalence (18.2%) as hypokalemia (**Table 3**). While hyperkalemia was rare throughout the study period, the prevalence of hypernatremia increased steeply during the first week of hospital stay (**Figure 2**). Hypernatremia was present in 1.5% of blood samples analyzed on day 0 (using, again, only the first daily sample per patient), compared to 23.0% of blood samples during week 2 (**Table 3**). Spot urine data were available in a subsample of nine patients in whom the urine K/Cr ratio increased during the hospital stay. While suggesting a weak negative correlation when plotted against serum potassium (**Figure 3**), the urine K/Cr ratio was above the cutoff value of 1.5 in only 3 (3.7%) of 81 total samples.

### C-reactive protein dynamics

**Figure 4:**
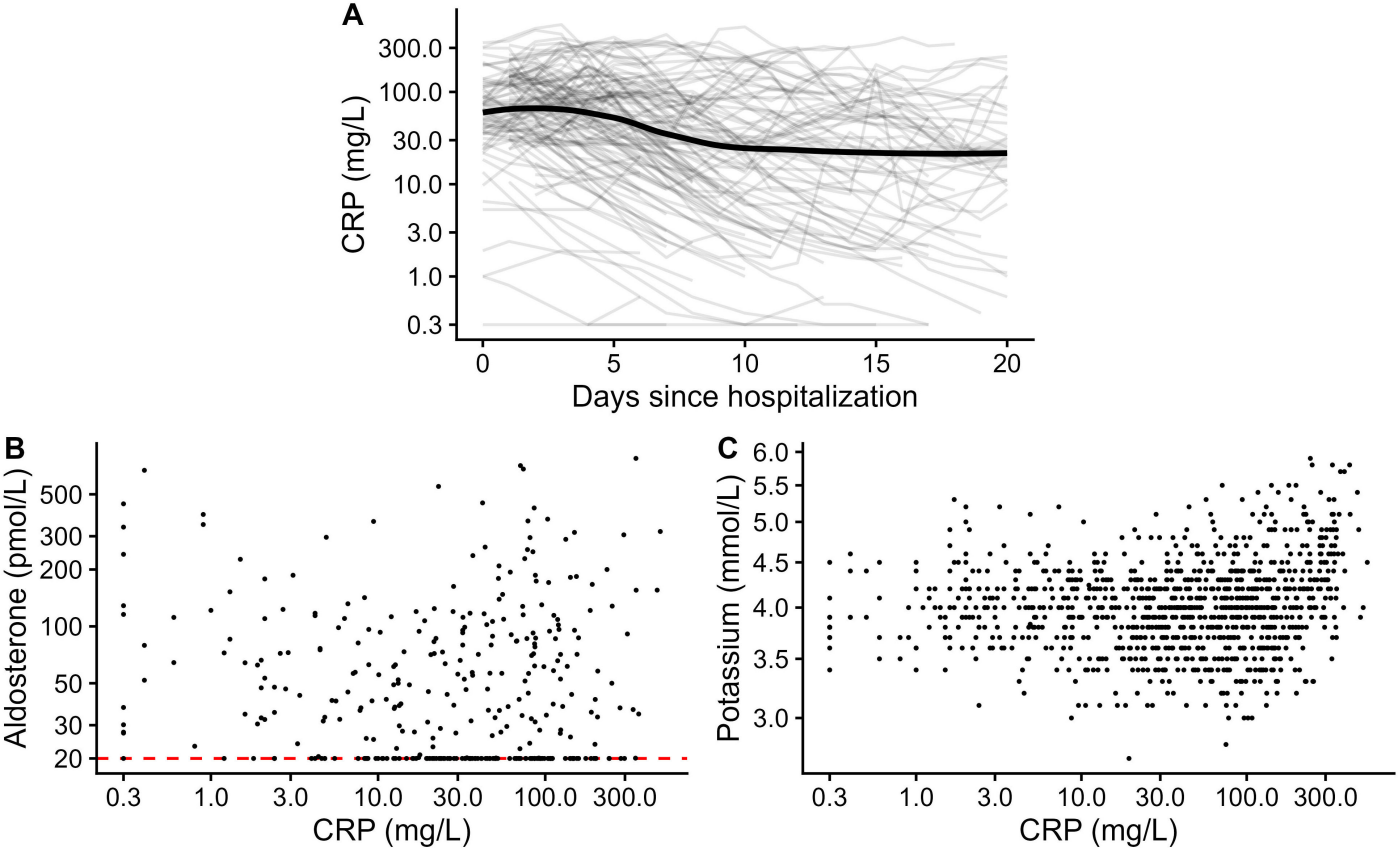
C-reactive protein over time and against aldosterone and potassium. C-reactive protein was depicted over time using LOESS smoothed curves with 95% confidence interval for the entire collective and as individual lines per patient in lighter gray **(A)**, scatter-plotted against plasma aldosterone **(B)**, and serum potassium levels **(C)** for all available first blood draws from the same day per patient. The lower limit of quantitation of aldosterone is indicated by a red dashed line. Axes for CRP, aldosterone, and potassium were depicted on the logarithmic scale. CRP, C-reactive protein.

The median of C-reactive protein was the highest during the first week of hospital stay, more than twice as high as in the following weeks [CRP in mg/L as median (IQR) in week 1: 76.9 (33.7, 145.7), in week 2: 32.2 (8.1, 101.2), in week 3: 30.0 (7.8, 79.9), **Table 3**]. On the population level, CRP showed a biphasic relationship, which appeared positive beyond a CRP of 100 mg/L, as illustrated in **Figure 4**. CRP beyond 100 mg/L was most prevalent during the first week of the analysis (week 1: 38.9%, week 2: 24.6%, week 3: 17.7% of the samples analyzed, **Table 4**).

**Table 3:** Potassium and sodium levels at first measurement during the first week of COVID-related hospital stay.

Variable	Female (*N* = 54)	Male (*N* = 105)	Overall (*N* = 159)
Potassium (mmol/L)	3.60 [3.4, 3.9]	3.80 [3.6, 4.1]	3.80 [3.5, 4.0]
Hypokalemia	13 (24.1%)	14 (13.3%)	27 (17.0%)
Normokalemia	25 (46.3%)	57 (54.3%)	82 (51.6%)
Hyperkalemia	1 (1.9%)	11 (10.5%)	12 (7.5%)
Missing	15 (27.8%)	23 (21.9%)	38 (23.9%)
Sodium (mmol/L)	139.0 [137.0, 141.0]	138.0 [134.0, 140.0]	138.0 [135.0, 141.0]
Hyponatremia	5 (9.3%)	24 (22.9%)	29 (18.2%)
Normonatremia	33 (61.1%)	55 (52.4%)	88 (55.3%)
Hypernatremia	1 (1.9%)	3 (2.9%)	4 (2.5%)
Missing	15 (27.8%)	23 (21.9%)	38 (23.9%)

Values are presented as either median and interquartile range for continuous variables or absolute and relative terms (%) for categorical variables. Missing values stem from patients who had no respective values recorded during the first week of stay.

**Table 4:** Bloodwork stratified by week.

Variable	Week 1	Week 2	Week 3
Total samples	452	382	243
Potassium (mmol/L), median [IQR]	3.9 [3.6, 4.2]	4.00 [3.8, 4.3]	4.00 [3.8, 4.3]
Hypokalemia, *N* (%)	64 (14.2)	22 (5.8)	16 (6.6)
Hyperkalemia, *N* (%)	17 (3.8)	16 (4.2)	6 (2.5)
Sodium (mmol/L), median [IQR]	140.0 [137.0, 143.0]	142.0 [139.0, 145.0]	141.0 [138.0, 145.0]
Hyponatremia, *N* (%)	57 (12.6)	10 (2.6)	12 (4.9)
Hypernatremia, *N* (%)	59 (13.1)	88 (23.0)	45 (18.5)
CRP (mg/L), median [IQR]	76.9 [33.7, 145.7]	32.20 [8.1, 101.3]	30.0 [7.8, 79.9]
CRP >100 mg/L, *N* (%)	176 (38.9)	94 (24.6)	43 (17.7)

Values are presented as either median and interquartile range for continuous variables or absolute and relative terms (%) for categorical variables stratified by in-hospital week.

CRP, C-reactive protein; IQR, bounds of the interquartile range.

### Relationship between potassium and aldosterone

GEE analyses were possible on 106 patients (GEE A) and 82 patients (GEEs B and C), respectively. Dynamic GEE analyses did not show any relevant or significant association of patient potassium levels with the most recent preceding blood aldosterone level. The beta coefficient (*β*) of the base-10 logarithm of aldosterone, its 95% confidence interval (95% CI), and *p*-value from GEE A were *β* = −0.008, 95% CI: −0.074 to 0.057, and *p* = 0.805, respectively (**Table 5**). This result was neither significantly changed by adding potassium-altering medication as covariates (GEE B with *β* = 0.013, 95% CI −0.064 to 0.090, *p* = 0.739, **Table 5**) nor by assuming predictor effects to decrease exponentially with time elapsed between the measurement of predictors and of the corresponding potassium (GEE C with *β* = 0.001, 95% CI: −0.078 to 0.075, *p* = 0.971, **Table 5**). All GEEs and descriptive analyses thus consistently failed to provide evidence for an association between aldosterone levels and change in potassium in our dataset. The full set of model parameters, beta coefficient estimates, 95% CIs, and *p*-values can be found in **Supplementary Tables S1**-**S3**; however, we generally advise against attempting to interpret these adjustment variable parameters to avoid a table 2 fallacy (31).

**Table 5:** GEE effect estimates of the most recent aldosterone on potassium.

Model	Estimate	95% CI	*P*-value
GEE A	−0.008	−0.074, 0.058	0.805
GEE B	0.013	−0.064, 0.090	0.739
GEE C	−0.001	−0.078, 0.075	0.971

Shown are effect estimates, 95% confidence intervals, and *p*-values of three adjusted generalized estimating equations for the logarithm to base 10 of the latest measured serum aldosterone levels. GEE A (700 observations from 106 patients) was adjusted for age, sex, current intensive care unit stay, time since hospitalization, latest serum potassium, and time interactions of time passed since measurements of the latest aldosterone and potassium, respectively. GEEs B and C (621 observations, 82 patients) were further adjusted for the latest angiotensin II receptor blocker, the latest angiotensin-converting enzyme inhibitor, the latest mineralocorticoid receptor antagonist, the latest loop diuretic, the latest thiazide drug, the latest potassium-flushing drug, the latest potassium supplementation, the latest catecholamine drug, and the respective interactions with time passed from their respective assessments to the assessment of potassium.

CI, confidence interval; GEE, generalized estimating equation.

### Relationship between angiotensin II and aldosterone

In GEEs with the DV aldosterone, Ang II was the strongest predictor variable. The addition of same-day potassium and ICU stay information as covariates did not significantly improve the estimated explained variance of aldosterone in these models (*R*² 0.265 versus 0.275, **Table 6**).

**Table 6:** Model fit comparison of generalized estimating equations with the dependent variable logarithm of aldosterone.

Model formula	Marginal *R*²	95% CI
log(Aldosterone) ~ log(Ang II)	0.265	0.147–0.405
log(Aldosterone) ~ potassium	0.009	<0.001–0.108
log(Aldosterone) ~ ICU	<0.001	<0.001–0.036
log(Aldosterone) ~ potassium + ICU	0.015	<0.001–0.101
log(Aldosterone) ~ log(Ang II) + potassium	0.259	0.137–0.405
log(Aldosterone) ~ log(Ang II) + ICU	0.271	0.163–0.400
log(Aldosterone) ~ log(Ang II) + potassium + ICU	0.275	0.171–0.403

Shown are bias-corrected marginal *R*² and bias-corrected, accelerated 95% confidence intervals derived from cluster-bootstrapped datasets using 5,000 resamples applied to separate generalized estimating equations with seven different combinations of predictors and model formulae in the style of the R programming language, denoting the model structures. The dependent variable is shown to the left, and the independent variables are shown to the right of the tilde symbol in these formulae.

Ang, angiotensin; CI, confidence interval; ICU, intensive care unit; log, logarithmic transformation to base 10.

## Discussion

The main findings of our study of hospitalized COVID-19 patients include unusually low aldosterone levels during hospitalization and no association of the most recent morning plasma aldosterone with change in potassium in any of the dynamic GEEs adjusting for relevant covariates, for medications, and for the exponential decay of effects of potassium over time. Increased aldosterone levels were therefore not a suitable explanation for potassium loss during the first 3 weeks of hospitalization in this cohort of patients with COVID-19 from the first wave of infections in central Europe, contrasting some of the previously published pathomechanistic assumptions (7).

The feedback loop between potassium and aldosterone and the exact onset of infection and disease are important factors to consider in the interpretation of these results (32). Serum potassium played only a very minor role in our cohort as an independent predictor in capturing the variance of aldosterone. The inclusion of serum potassium as a covariate in the model even decreased variance capture in GEEs with Ang II as a predictor, if ICU stay information was also not included. This finding may be a consequence of the observation interval being set after hospitalization, including stringent potassium resuscitation in the ICU. It is further possible that potassium loss through alternate routes curbs hyperaldosteronism or that aldosterone is higher before hospitalization. An unstructured subsample of nine patients with repeat spot urine tests available by clinical indication showed relatively low urine K/Cr ratio values (96.3% of samples below 1.5) and was not indicative of excessive urinary potassium loss during the in-hospital period.

Our finding of higher hypokalemia rates in women ties in well with previous studies: Pani et al. similarly observed rates in women that were twice as high as in men (33), and a 1.68-fold rate in women can be deduced from the data reported by Alfano et al. (34). Female predisposition regarding hypokalemia is also documented outside of COVID-19 (35) and may be connected to the systematically lower potassium stores in women (36).

Previous reports of aldosterone levels in COVID-19 are scarce. One study found that aldosterone concentrations were below the lower limit of quantitation of 70 pmol/L in 58.7% of the first available blood sample of patients with COVID-19 when using liquid LC/MS-MS (the same method used in the present study), which alarmingly were not correctly detectable by a more conventional non-extraction immunoassay method, probably due to interference with aqueous solutes (37). In our study using LC/MS-MS with a more sensitive lower limit of quantitation of 20 pmol/L, aldosterone levels were still below the limit of quantitation in 32.4% of the samples. This finding is in favor of secondary hypoaldosteronism, potentially as a consequence of potassium depletion, rather than potassium loss through ACE2-mediated increased aldosterone as previously proposed.

The original hypothesis of ACE2 depletion causing potassium loss during COVID-19 appears plausible and straightforward on a mechanistic level. During infection, SARS-CoV-2 uses membrane-bound ACE2 for cell entry (38). ACE2 is the key component of the alternative axis of the RAAS, converting Ang I to Ang 1–9 and Ang II to Ang 1–7, effectively lowering Ang II levels (39). Ang II stimulates the release of aldosterone, which in turn lowers serum potassium (39), and serum potassium itself is part of a feedback loop system with aldosterone (32).

Empirically supportive of the theory of ACE2 depletion and resulting aldosterone-mediated potassium loss, one study “in some cases, [ … ] observed a trend toward an association between higher levels of aldosterone and lower renin and potassium levels” and found higher aldosterone levels to be associated with severity of disease in COVID-19 (40). In another study, upward trajectories of ACE2 were reported to be associated with increased mortality, while ACE2 levels per se were not (41). While the high rates of increased kaliuresis with concomitant low urinary sodium concentration found in COVID-19 patients do fit the RAAS-mediated potassium loss theory, they are confounded by diuretic and corticosteroid therapy and also compatible with other potential causes of urinary potassium loss such as proximal tubular dysfunction, for which there is strong histopathological evidence (25–27). The epithelial Na^+^ channel was further found to be activated directly by SARS-CoV-2 nucleocapsid protein in murine models, leading to potassium loss without involvement of the RAAS cascade (28). In summary, there are multiple alternative routes for urinary potassium loss during COVID-19, which do not involve mediation by RAAS or ACE2. In spite of the aforementioned and regardless of the lack of direct evidence, the simple and rarely challenged theory of ACE2 depletion and increased aldosterone remains highly prevalent in the literature on renal potassium loss in COVID-19 to this day (2, 6, 16–20). Our data did not support this theory during the in-hospital period, fundamentally challenging these popular etiological assumptions.

### Strengths and limitations

The present study is the first to directly investigate the association of aldosterone with changes in potassium levels in COVID-19. We had a sufficient sample size, carried out multiple sensitivity analyses, and descriptively explored changes in relevant parameters over time. The study was limited by the fact that the dataset was not originally collected for the purpose of this investigation, involved mainly male patients, and did not include screening of urine potassium levels. Furthermore, we measured systemic RAAS activity and potassium in the patients’ blood and therefore cannot account for local tissue concentrations and effects. While adjusting for multiple covariates, we were unable to assess and include in our models all possible confounding variables, such as the volume of the most recent intravenous fluid infusions and adrenal insufficiency. Samples for aldosterone measurements were taken in the morning in the supine position, which is standard practice, but this cannot optimally represent variation throughout the day. Data regarding circadian rhythm, acid–base balance, corticosteroid use, feeding state, and timing of medications were not available in this study and could further influence the observed dynamics between potassium and aldosterone. The complex influences of these factors require separate analyses beyond the scope and setting of our analysis. It should be considered that individual differences in these factors are subsumed in the total result and may have attenuated the observable effect. Despite statistical adjustment for relevant medications, some residual confounding by indication remains possible. We were only able to consider medications and fluid supplementation on a binary level per day, potentially reducing the precision of GEEs. The excretory mechanism was not confirmed by this study. The observational setting at a single center during the first wave of infections in central Europe may limit generalizability. Data did not cover the prehospital infection period, and the initial potassium levels at infection onset remain unknown. Data were also sparser during the last week of the observation interval.

## Conclusion

Systemic aldosterone was low during 3 weeks of COVID-19-associated hospital stay and was not associated with serum potassium levels. This finding does not align with the untested, yet oft-cited, mechanistic theory of renal potassium loss via increased aldosterone levels mediated by ACE2 depletion. Future analyses should aim to cover the prehospitalization period, to record all potentially relevant factors in the aldosterone–potassium relationship, and to generate evidence capable of directly weighing this ACE2 depletion theory against competing theories of renal potassium loss in COVID-19, like proximal tubular dysfunction and direct nucleocapsid-based activation of ENaC.

## Data Availability

Due to the number of participants and identifiable time frame and location of the study, data has not been made publicly available to protect privacy rights. The data used in this study can be made available upon request from the corresponding author after conferring with ACOVACT study principal investigator. Requests to access these datasets should be directed to manfred.hecking@meduniwien.ac.at.
