# Supplementary material for: Plasma aldosterone is low in patients hospitalized with COVID-19 and not associated with changes in serum potassium levels: post hoc observational analyses of clinical trial data
Source: Front Endocrinol (Lausanne). 2025 Dec 10;16:1706679. doi: 10.3389/fendo.2025.1706679 (PMC12727438; doi:10.3389/fendo.2025.1706679)
Supplement: Supplementary file 1 [file DataSheet1.pdf]

Plasma aldosterone is low in patients hospitalized with COVID-19 and not associated with changes in serum potassium levels: post-hoc observational analyses of clinical trial data

Supplementary Material

Supplementary Table S1. Generalized estimating equations of serum potassium.

Term	Estimate	95%-CI	p-Value
Intercept	1.40150	1.07276,1.73024	<0.00001
Age	0.02015	0.00653,0.03376	0.00372
Sex [m]	0.02139	-0.06633,0.10911	0.63265
ICU	0.03243	-0.03769,0.10255	0.36473
Days in Hospital	-0.00203	-0.00813,0.00406	0.51373
Logarithm of Latest Aldosterone	-0.00830	-0.07425,0.05765	0.80522
Latest Potassium	0.63042	0.54415,0.71670	<0.00001
Latest Aldosterone Interaction with Elapsed Time	0.00051	-0.00507,0.00609	0.85775
Latest Potassium Interaction with Elapsed Time	-0.00332	-0.01128,0.00463	0.41305

Shown are effect coefficients, 95% confidence intervals, p-values, (700 observations from 106 patients). Independent variable of interest was the logarithm to base 10 of latest measured serum aldosterone levels. Models were adjusted for age, sex, current intensive care unit stay, time since hospitalization, latest serum potassium, and time interactions of time passed since measurements of the latest aldosterone and potassium respectively. Abbreviations: CI: confidence interval; ICU: intensive care unit; m: male.

Supplementary Table S2. Generalized estimating equations of serum potassium using comedications as independent variables.

Term	Estimate	95%-CI	p-Value
Intercept	1.51543	1.18975,1.84112	<0.00001
Age	0.02534	0.00764,0.04304	0.00502
Sex [m]	0.00668	-0.08285,0.09622	0.88366
ICU	-0.00204	-0.07783,0.07375	0.95787
Days in Hospital	-0.00561	-0.01455,0.00334	0.21904
Logarithm of Latest Aldosterone	0.01316	-0.06418,0.09049	0.73879
Latest Potassium	0.59306	0.50604,0.68008	<0.00001
Latest ARB	-0.09787	-0.18822,-0.00753	0.03374
Latest ACEi	-0.05270	-0.15730,0.05190	0.32340
Latest MRA	0.03024	-0.07835,0.13883	0.58516
Latest Loop Diuretic	-0.20533	-0.30381,-0.10685	0.00004
Latest Thiazide Diuretic	-0.18473	-0.37364,0.00418	0.05529
Latest Potassium Flushing Drug	0.04414	-0.22891,0.31720	0.75135
Latest Potassium Supplementation	0.04375	-0.03983,0.12733	0.30492
Latest Catecholamines	0.17564	0.09545,0.25582	0.00002
Latest Aldosterone Interaction with Elapsed Time	0.00017	-0.00558,0.00592	0.95303
Latest Potassium Interaction with Elapsed Time	-0.00093	-0.00953,0.00768	0.83281
Latest ARB Interaction with Elapsed Time	0.01265	0.00351,0.02179	0.00668
Latest ACEi Interaction with Elapsed Time	0.00490	-0.00444,0.01424	0.30377
Latest MRA Interaction with Elapsed Time	0.02749	-0.00421,0.05920	0.08921
Latest Loop Diuretic Interaction with Elapsed Time	0.00665	-0.00558,0.01887	0.28650
Latest Thiazide Diuretic Interaction with Elapsed Time	0.00861	-0.01673,0.03395	0.50539
Latest Potassium Flushing Drug Interaction with Elapsed Time	0.01610	-0.02722,0.05942	0.46641
Latest Potassium Supplementation Interaction with Elapsed Time	-0.00252	-0.01316,0.00812	0.64217
Latest Catecholamine Interaction with Elapsed Time	0.00932	-0.00475,0.02338	0.19407

Shown are effects, 95% confidence intervals, p-values, number of included patients and number of total observations of the independent variable of interest (621 observations from 82 patients). Independent variable of interest was the logarithm to base 10 of latest measured serum aldosterone levels, adjusted for age, sex, current intensive care unit stay, time since hospitalization, latest serum potassium, and time interactions of time passed since measurements of the latest aldosterone and potassium respectively, latest angiotensin II receptor blocker, latest angiotensin converting enzyme inhibitor, latest mineralocorticoid receptor antagonist, latest loop diuretic, latest thiazide drug, latest potassium flushing drug, latest potassium supplementation, latest catecholamine drug and respective interactions with time passed from their respective assessments to the assessment of potassium. Abbreviations: ACEi: angiotensin converting enzyme inhibitor; ARB: angiotensin II receptor blocker; CI: confidence interval; m: male; MRA: mineralocorticoid receptor antagonist.

Supplementary Table S3. Generalized estimating equations of serum potassium, modelling exponential effects decrease over time.

Term	Estimate	95%-CI	p-Value
Intercept	1.67869	1.33180,2.02558	<0.00001
Age	0.02197	0.00321,0.04073	0.02169
Sex [m]	0.01108	-0.07714,0.09930	0.80555
ICU	0.01201	-0.07498,0.09899	0.78675
Days in Hospital	-0.00426	-0.01214,0.00363	0.29002
Logarithm of Latest Aldosterone	-0.00143	-0.07828,0.07541	0.97084
Latest Potassium	0.55665	0.46858,0.64472	<0.00001
Latest ARB	-0.14770	-0.26753,-0.02786	0.01570
Latest ACEi	-0.09317	-0.23064,0.04430	0.18406
Latest MRA	0.01240	-0.11742,0.14221	0.85152
Latest Loop Diuretic	-0.19775	-0.30045,-0.09505	0.00016
Latest Thiazide Diuretic	-0.33198	-0.51601,-0.14795	0.00041
Latest Potassium Flushing Drug	-0.03799	-0.47083,0.39485	0.86341
Latest Potassium Supplementation	0.01350	-0.07478,0.10179	0.76437
Latest Catecholamines	0.16938	0.08654,0.25223	0.00006
Latest Aldosterone Interaction with Elapsed Time	0.04253	-0.07160,0.15665	0.46518
Latest Potassium Interaction with Elapsed Time	-0.04082	-0.11458,0.03294	0.27811
Latest ARB Interaction with Elapsed Time	0.37366	0.10498,0.64233	0.00641
Latest ACEi Interaction with Elapsed Time	0.22808	-0.10161,0.55778	0.17512
Latest MRA Interaction with Elapsed Time	0.37374	-0.02155,0.76904	0.06386
Latest Loop Diuretic Interaction with Elapsed Time	-0.04228	-0.21307,0.12851	0.62756
Latest Thiazide Diuretic Interaction with Elapsed Time	0.45874	0.00195,0.91553	0.04903
Latest Potassium Flushing Drug Interaction with Elapsed Time	0.43234	-0.58348,1.44816	0.40418
Latest Potassium Supplementation Interaction with Elapsed Time	0.08852	-0.09532,0.27236	0.34528
Latest Catecholamine Interaction with Elapsed Time	0.01317	0.00016,0.02617	0.04721

Shown are effects, 95% confidence intervals, p-values, number of included patients and number of total observations of the independent variable of interest (621 observations from 82 patients). Independent variable of interest was the logarithm to base 10 of latest measured serum aldosterone levels, adjusted for age, sex, current intensive care unit stay, time since hospitalization, latest serum potassium, and time interactions of time passed since measurements of the latest aldosterone and potassium respectively, latest angiotensin II receptor blocker, latest angiotensin converting enzyme inhibitor, latest mineralocorticoid receptor antagonist, latest loop diuretic, latest thiazide drug, latest potassium flushing drug, latest potassium supplementation, latest catecholamine drug and respective interactions with time passed since their respective assessments to the assessment of dependent variable potassium. In analysis the time since the assessment of all covariates was transformed to reflect exponential decrease of effects over time passed between their assessment and the assessment of the dependent variable potassium. Abbreviations: ACEi: angiotensin converting enzyme inhibitor; ARB: angiotensin II receptor blocker; CI: confidence interval; GEE: generalized estimating equations; ICU: intensive care unit; m: male; MRA: mineralocorticoid receptor antagonist.

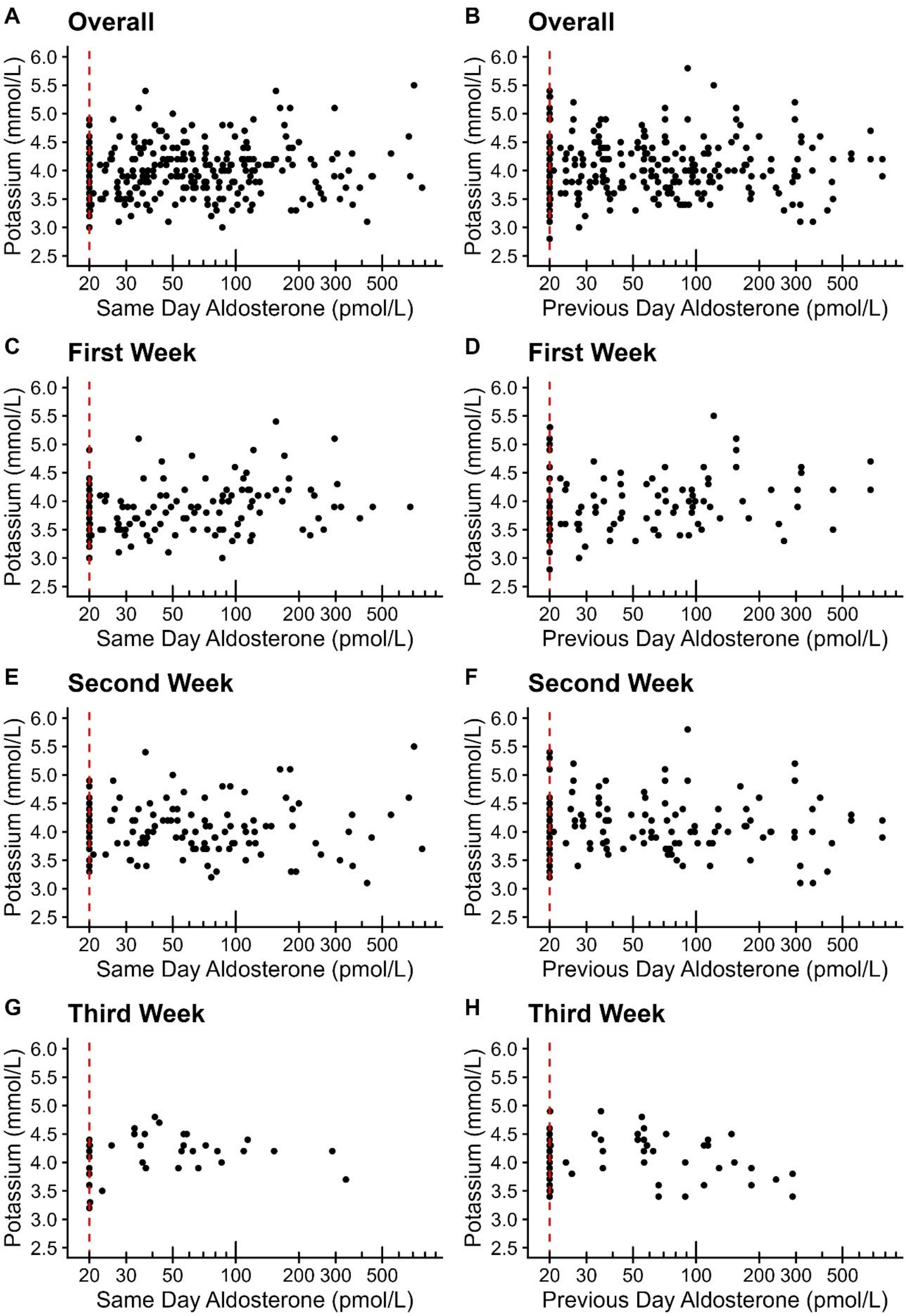

Supplementary Figure S1. Scatter plots of potassium and aldosterone. Plots on the left (A, C, E, G) show aldosterone at the same day as potassium, while plots on the right (B, D, F, H) show aldosterone of the previous day if it was measured. Rows correspond to the timeline, A and B showing the full data from the entire 3-week observation period, B and C showing only potassium data from the first week of hospital stay, D and E showing the second week, and G and H showing the third week. The dashed lines indicate the lower level of quantification for aldosterone. Horizontal axes are logarithmically scaled.

Full Inclusion and Exclusion Criteria of the Underlying Trial

All data of the present study were supplied from the Austrian COronaVirus Adaptive Clinical Trial (ACOVACT), which was registered with clinicaltrials.gov (ClinicalTrials.gov identification code NCT04351724) and the European Union Drug Regulating Authorities Clinical Trials Database (EudraCT identification code 2020-001302-30) and had the following inclusion and exclusion criteria.

Inclusion Criteria

- *Laboratory confirmed (i.e. PCR-based assay) infection with SARS-CoV-2*
- *Hospitalisation due to SARS-CoV-2 infection*
- *Requirement of oxygen support (due to oxygen saturation <94% on ambient air or >3% drop in case of chronic obstructive lung disease)*
- *Informed Consent obtained, the patient understands and agrees to comply with the planned study procedures, except for sub-study C: obtaining informed consent may be impossible due to the severe condition of the patient and may be waived*
- *≥18 years of age*
- *Sub-study A: not on chronic anticoagulation Sub-study B: Sub-study B: blood pressure ≥130/85mmHg in 2 consecutive measurements OR patients with established and treated hypertension*
- *Sub-study B: Control group 1: Patients with suspicion of, but negative tests for, COVID-19. This group may consist of hospitalized and non-hospitalized patients.*
- *Sub-study B: healthy volunteers*
- *Sub-study C: Signs of respiratory deterioration and progressing inflammation: need for oxygen supplementation, non-invasive ventilation, high-flow oxygen devices or mechanical ventilation AND CRP levels >5mg/dL (for Pentaglobin only) and ICU admission (for Pentaglobin only)*
- *For female patients with childbearing potential: willingness to perform effective measures of contraception during the study*

Exclusion Criteria

- *Moribund, or estimated life expectancy <1 month (e.g. terminal cancer, etc.)*
- *Patient does not qualify for intensive care, based on local triage criteria*
- *Pregnancy or breastfeeding*
- *Severe liver dysfunction (e.g. ALT/AST > 5 times upper limit of normal)*
- *Stage 4 chronic kidney disease or requiring dialysis for direct anticoagulant treatment*

- *Allergy or intolerances to experimental substance (ineligibility for treatment arm), for Asunercept known hereditary fructose intolerance*
- *Anticipated discharge from hospital within 48 hours (for any given reason)*
- *Contraindications for treatment arm 2 (lopinavir/ritonavir): severe hepatic impairment, CYP3A4/5 metabolized drugs, as deemed relevant by treating physicians*
- *Contraindications for treatment arm 3 (remdesivir): <40kg bodyweight*
- *Known active HIV or viral hepatitis*
- *Substudy A contraindications for rivaroxaban: active bleeding or bleeding diathesis, lesion or condition considered as major risk factor for bleeding, recent brain or spinal injury, recent brain or spinal or ophthalmic surgery, recent intracranial hemorrhage, known or suspected esophageal varices, arteriovenous malformations, vascular aneurysms, major intraspinal or intracerebral vascular abnormalities, ongoing therapeutic anticoagulation, which will be continued, according to clinical practice*
- *Sub-study B contraindications for nitrendipine: chronic heart failure, allergies, hypersensitivities and intolerances, severe hepatic impairment and/or cholestasis, concomitant therapy with aliskirencontaining medications (for patients with diabetes mellitus or a GFR<60ml/min/1.73m2), known significant bilateral renal artery stenosis or renal artery stenosis of a solitary kidney*
- *Sub-study C contraindications for IL-6 blockade: Contraindications: allergies and intolerances, active untreated diverticulitis, inflammatory bowel disease, any treatment with an IL-6 or IL-6R blocking drug (e.g. tocilizumab, sarilumab, siltuximab) <30 days before study inclusion.*
- *Sub-study C: Known active tuberculosis.*
- *Asunercept: females of childbearing potential*
- *Sub-study C with Pentaglobin: Contraindications to Pentaglobin*
